# Association Pathways Between Neighborhood Greenspaces and the Physical and Mental Health of Older Adults—A Cross-Sectional Study in Guangzhou, China

**DOI:** 10.3389/fpubh.2020.551453

**Published:** 2020-09-22

**Authors:** Yuquan Zhou, Yuan Yuan, Yujie Chen, Shulin Lai

**Affiliations:** ^1^Guangdong Key Laboratory for Urbanization and Geo-simulation, School of Geography and Planning, Sun Yat-sen University, Guangzhou, China; ^2^Department of City and Regional Planning, College of Environmental Design, University of California, Berkeley, Berkeley, CA, United States

**Keywords:** neighborhood greenspace, physical health, mental health, older adult, structual equation model, Guangzhou

## Abstract

According to the United Nations, the proportion of the older population is increasing at a faster rate than all other age groups. Hence, the well-being of older adults is a mounting concern worldwide in the current century. Using a single greenery metric, previous studies linked greenness to residents' well-being. This study aims to extend this field by focusing on the mental and physical well-being of older adults by using remote sensing and streetscape metrics in evaluating neighborhood greenness. We selected 20 residential neighborhoods in Guangzhou City, China as the cross-sectional case study areas. We investigated neighborhood normalized difference vegetation index (NDVI) collected using remote sensing images, streetscape greenery, and PM2.5 via field surveys. We assessed the health condition of 972 senior residents selected by multi-stage stratified probability proportionate to population size sampling technique (PPS) using a questionnaire survey. We adopted the structural equation model (SEM) in analyzing the pathways that link neighborhood greenness and the mental and physical health of older adults. We found that neighborhood greenness has a positive association with the physical activity by older adults that is positively linked to their physical health. Moreover, neighborhood greenness is positively related to regular social interactions among older adults that is positively linked to their mental health. These findings are consistent with those of previous studies. However, we obtained new results that were unique to China. We found that neighborhood greenness has no significant direct relationship with the physical and mental health of older adults and that social interactions of low-income senior groups are more substantially related to neighborhood greenness than the other groups. Therefore, community planning should emphasize the development of neighborhood greenness, such as parks and street trees, to provide natural spaces for social interactions and places for physical activities among older residents.

## Introduction

The 21st century is an era characterized by aging and urbanization, and these characteristics are more prominent in developing countries. According to the World Health Organization (WHO), the proportion of seniors (aged 60 years and older) to the global population will reach 22% in 2050 (1). Both the aging rate (Proportion of Population ages 65 and above) (2) and urbanization rate (Proportion of Urban Population) (3) in China, are higher than the global average. Improving the physical and mental health of the older adults in urban areas has become an important issue in China.

Numerous studies conducted in developed countries have demonstrated that greenspace exposure is related to wide-ranging health benefits, including better mental health and physical health (4–15). In terms of mental well-being, exposure of residents to greenspaces may enhance their feelings of happiness and relieve their stress from negative events (16, 17). In terms of physical well-being, exposure to greenspaces has an active role in reducing morbidity from multiple diseases (18–20). Several studies that focused on greenspaces in China explored the relationship between neighborhood environment and residents' well-being (21–25), which reported positive relationship between neighborhood greenspaces and residents' well-being, especially in terms of mental health.

Overall, most studies associating greenspaces and health have been conducted in developed countries. By contrast, other studies in developing countries, such as China, have focused particularly on relationship between greenspaces and residents' mental health using just a single greenness metrics, which could have possibly resulted in biased estimations of indicators (26). In addition, in the face of growing older population, research on the association between neighborhood greenery and older adults' mental and physical well-being is relatively lacking.

This study aimed to address this gap and conducted a cross-sectional empirical research using survey data collected from 20 neighborhoods in Guangzhou City, China, a highly populated city characterized by rapid urbanization and a large proportion of immigrant populations (27), to explore the pathways that link neighborhood greenspaces and older individuals' physical and mental well-being. This study makes the following contributions to knowledge on this topic. First, it focused particularly on older adults in China and used multidimensional survey questions to assess older adults' mental and physical health status to disentangle the aging issues from neighborhood greenspace perspective. Second, both neighborhood normalized difference vegetation index (NDVI) from bird's eye-view and streetscape greenery from human eye-level metrics were measured to quantify neighborhood greenery well. Third, it adopted the multigroup structural equation model in exploring the differences in pathways among older adults with different demographic backgrounds.

## Literature Review

The pathway mechanism between greenspaces and health includes direct and mediating pathways that may be different among individuals with different sociodemographic backgrounds.

Numerous studies have revealed the pathway that greenspace exposure directly relates to residents' mental and physical health. In terms of physical health, green in the middle of the color spectrum is more beneficial to human health, especially to the brain and the nervous system than the other colors (28). Moreover, empirical research have shown that natural environments can effectively alleviate headaches by 52% (29). In terms of mental health, visually seeing greenery or green plants alone can help relieve tension and anxiety, and inhaling plants' essential oils can induce changes in psychological state, thereby affecting the psychological stability of the human body (30–33). Given that natural environments are less complicated than urban environments, greenery is conducive in reducing an individual's stress levels and in restoring attention (34–37). These findings have been verified by multiple empirical studies in China (21, 22) and in other developed countries, such as the Netherlands (38) and the United States (39).

In terms of mediating pathways, mediators, such as air pollution, social interactions, and physical activities, also mediate the association between neighborhood greenspaces and residents' well-being. Air pollution, such as nitrogen dioxide, fine particulate matter (such as PM_2.5_), and ozone, has negative health effects, and many studies have demonstrated the negative association between surrounding greenspaces and air pollution (40–42). Greenspaces can help in mitigating urban microclimates and effectively reducing urban environmental pollution and filtering health-threatening air pollutants by sticking wind-blown particulates, such as PM_2.5_ and PM_10_, to plant leaves and stems (43–45). Meanwhile, long-term exposure to air pollution such as PM2.5 is related to increased all-cause and cardiopulmonary mortality (46, 47), as well as mental disorders (48, 49). An empirical research conducted in Toronto, Canada showed that green roofs on downtown buildings contribute positively to the health of citizens via the air pollution mitigation (50). In summary, greenspaces can effectively improve air quality, and thereby ameliorating residents' health.

The second mediating pathway is via physical activities. Neighborhood greenspaces can be used as a space for physical activities, such as walking, jogging, or cycling, for residents. Greenspaces positively link to individuals' healthy behavior by encouraging them to do physical activities (51, 52). Meanwhile, physical activities benefits health and well-being of individuals from all ages (53). Furthermore, physical exercises performed in greenspaces may produce more health benefits than when done in other environments (54, 55), and limited greenspaces are positively related to sedentary lifestyle, which increases the risks of cardiovascular diseases due to obesity (56). Therefore, the greenspaces could positively link to positive health outcomes via physical exercise.

The third mediating pathway is associated with social interactions. Studies have shown that exposure to greenery may facilitate neighborhood social interactions that may foster the residents' well-being (21, 38, 57). Since greenspace may function as a place for social interactions, it may act as an intermediary variable that links the green environment to residents' health, promote social cohesion by providing a meeting place where people can engage in community activities (58, 59), and help residents obtain social support and reduce feelings of loneliness, thereby reducing stress and fatigue. Such spaces have an indirect positive relationship with mental health (60). Neighborhood greenspaces are particularly essential to aging generation because seniors are generally less mobile and have limited activity spaces and smaller social networks than the other age groups (59). In addition, harmonious social relationships, especially good neighborhood relationships, can promote residents' physical well-being (61, 62).

Based on this literature review, the hypothesis of this study is that neighborhood greenspaces have a direct or indirect linking path with the physical and mental health of the older adults. On the one hand, neighborhood greenspaces directly associate with older adults' physical and mental health. On the other hand, neighborhood greenspaces positively relate to older adults' physical and mental health via neighborhood air quality and older adults' physical exercise and social interaction.

**Figure 1:**
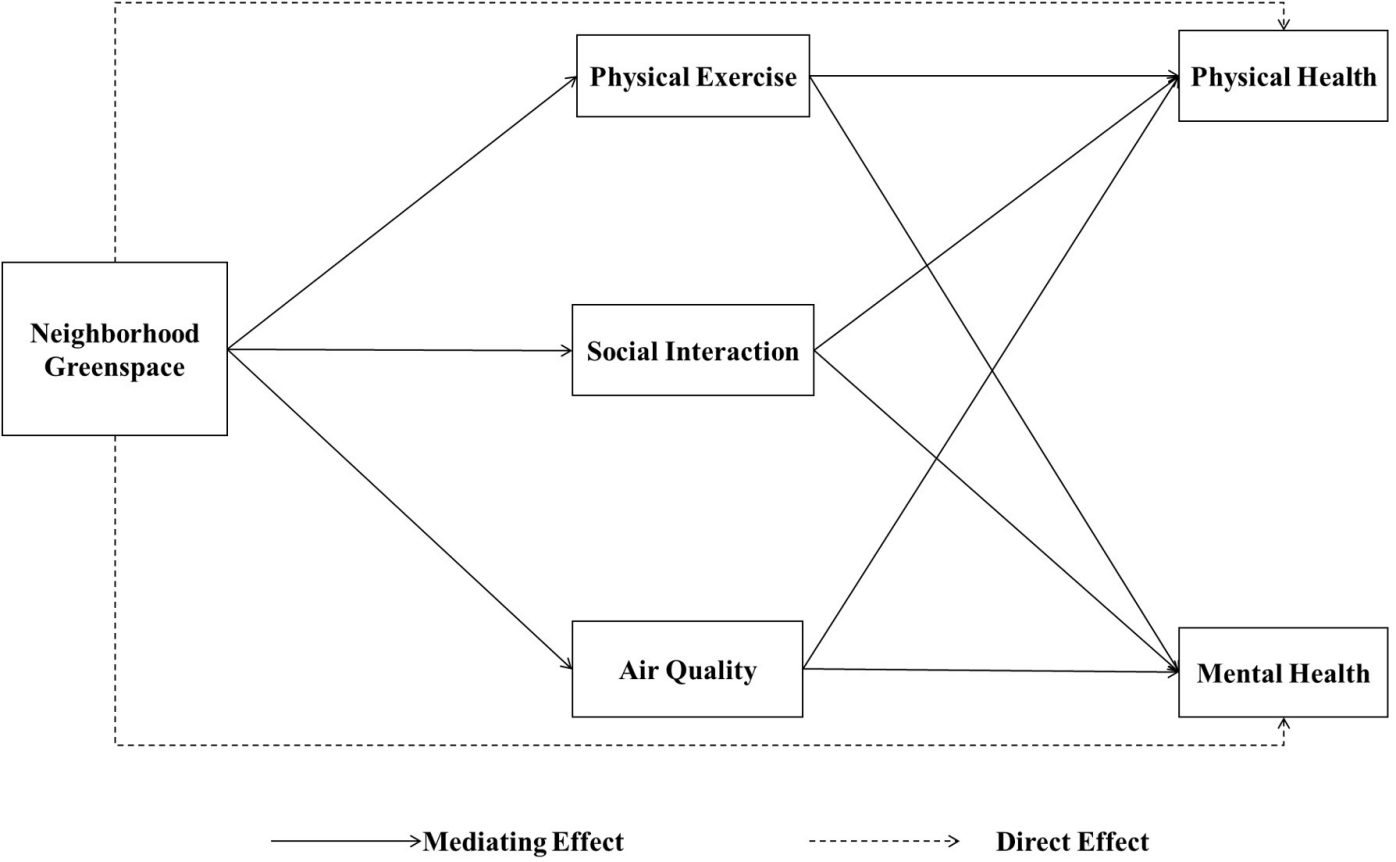
Theoretical pathways of the study.

The theoretical structural equation model below was built on the basis of these hypotheses (Figure 1).

However, this association between neighborhood greenspace and health may differ among individuals with different socio-demographic characteristics, since they have various opportunities and motivations to access greenspace (60). In terms of income, studies have shown that low-income individuals are more sensitive to greenspace exposure (63), since low-income communities are more likely to have limited access to green spaces (64). As for the age, multiple researches have shown that the health status and health related behaviors of older adults are relatively more related to neighborhood greenspace than other age groups (19, 65, 66), since they tend to spend more time in the communities (67). Regarding gender, since there are gender differences in perceptions and usage of urban green spaces, the health of female individuals are more related to greenspaces than males (68). However, there are few studies focused on the association differences among individuals with different marriage and registered residence status (hukou).

## Study Design

### Data Source and Characteristics

#### Study Area and Survey Data

**Figure 2:**
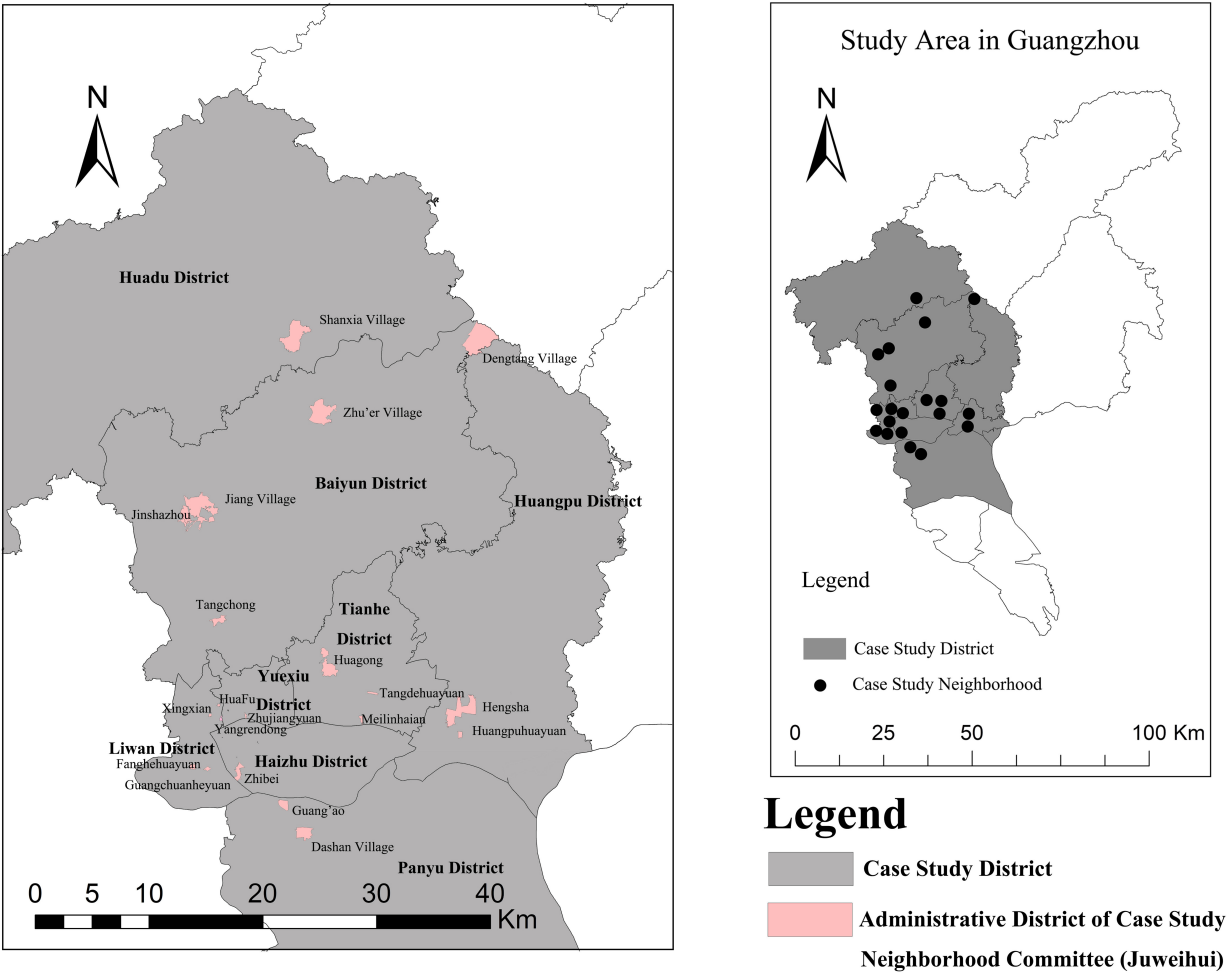
Locations and administrative boundaries of the 20 neighborhoods in Guangzhou City, China sampled in this study.

A multi-stage stratified probability proportionate to population size sampling technique (PPS) was adopted to select respondents. First, on the basis of the Sixth National Population Census data in China and previous research (69), Guangzhou was divided into six types of social areas of older adults as shown in Table 1. Subsequently, 19 streets (jiedao) from these six social areas were selected, focusing on areas with the highest score on factors of interest, and 20 case study neighborhoods were chosen with more than 10% elderly populations (aged 60 and older). The neighborhoods covered six different housing types in Guangzhou City: historic housing, institutional housing, affordable housing, rural self-built housing, commercial housing, and urban village housing (Table 1, Figure 2). Second, with the number of questionnaires in each neighborhood based on the percentage of its older adults population, a total of 972 valid questionnaire surveys of randomly selected residents who had lived in Guangzhou for over 6 months and aged 60 and older were conducted by a trained interviewer via face-to-face interview from December 2018 to April 2019. All respondents involved in this study gave their informed consent, and our study has been approved by institutional review board of school of geography and planning, Sun Yat-sen University. The questionnaire covered information on individuals' economic and social attributes, physical and mental health status, physical activity, and social interactions.

**Table 1:** Geographical characteristics and sample size of the 20 case study neighborhoods.

**Social areas of older adults**	**District**	**Street (jiedao)**	**Neighborhood**	**Housing types**	**Sample size**
Concentrated distribution areas of older adults in old neighborhood	Yuexiu	Zhuguang	Zhujiangyuan	Historic housing	72
	Liwan	Lingnan	Yangrendong	Historic housing	28
		Hualin	Xingxian	Historic housing	28
		Longjing	Huafu	Historic housing	10
Concentrated distribution areas of retired older adults in government agencies, enterprises, and institutions	Liwan	Baihedong	Guangchuanheyuan	Institutional Housing	110
	Haizhu	Nanshitou	Zhibei	Institutional Housing	128
	Tianhe	Yuancun	Meilinhaian	Commercial housing	36
	Huangpu	Huangpu	Huangpuhuayuan	Commercial housing	32
Scattered distribution area of retired elderly in education and scientific research units	Tianhe	Wushan	Huagong	Institutional Housing	94
Concentrated distribution areas of older adults in suburban rural areas in urban setting	Baiyun	Zhongluotan	Dengtang Village	Rural self-built housing	52
			Zhuer Village	Rural self-built housing	35
		Jianggao	Jiang Village	Rural self-built housing	21
	Huadu	Huadong	Shanxia Village	Rural self-built housing	49
Mixed population distribution area	Baiyun	Jinsha	Jinshazhou	Affordable housing	92
	Liwan	Dongjiao	Fanghehuayuan	Affordable housing	22
	Panyu	Luopu	Guang'ao	Commercial housing	23
	Huangpu	Dasha	Hengsha	Urban village housing	32
Concentrated new development areas of younger generation	Tianhe	Tangxia	Tangdehuayuan	Affordable housing	8
	Baiyun	Xinshi	Tangchong	Urban village housing	44
	Panyu	Dashi	Dashan Village	Urban village housing	56

#### Greenspace Data

We acquired streetscape greenery data and NDVI to measure the amount of greenery from street and overhead views in each neighborhood, respectively. The streetscape greenery data were gathered via field surveys in these neighborhoods from March 2019 to April 2019. The data were from obtained from digital photographs taken from sampling points and calculated using the “Maoyanxiangxian” streetscape greenery calculation application. The sampling points were 20 m apart and identified along roads and alleys in and around the neighborhoods from 0, 90, 180, and 270°facing north at a normal view of a human (1.6 m) (70, 71). A total of 2,544 street view images were collected from 636 sampling points.

The satellite-based NDVI (72) of each neighborhood was calculated on the basis of 1,000 m buffer around the boundary of the administrative district of Guangzhou Community Neighborhood Committee and Landsat 8 Operational Land Imager Thermal Infrared Sensor satellite remote sensing image at a 30 × 30 m spatial resolution in October 2017 with only 0.05 cloud cover using Formula 1 from Geospatial Data Cloud (http://www.gscloud.cn) (73). NDVI was calculated as follows:

$$\begin{array}{l} \text{NDVI}=(\text{NIRband}5\text{ - Redband}4)/\;(\text{NIRband}5+\text{Redband}4). \end{array}$$

#### Mediators

Data on mediators, including degree of air pollution in each neighborhood physical activity and social interactions, of older adults are were acquired via field surveys and questionnaires.

The level of physical activity of each older adult was determined by the average time spent on physical exercises, such as walking, per day from the questionnaire survey. The unit of measurement was hour.

Social cohesion can be defined in several ways. In this study, we focused on relatively weak social ties of community network. The level of social interaction was determined by asking each senior on what level they agree with the statements that “I know many people in the community” and “I am willing to communicate with community members.” The five categories of responses were “Strongly agree,” “Agree,” “Not decided,” “Disagree,” and “Strongly Disagree” and coded into 5–1, respectively. The social interaction variable was treated as a latent variable.

We used PM_2.5_ concentrations obtained in each neighborhood to assess the seniors' exposure to air pollution and recorded at the same sampling points with streetscape greenery. The outcome was calculated from the average of PM_2.5_ concentration in each neighborhood.

### Analysis Method

Multiple studies explored the pathway between greenery and residents' health by adopting multi-quantitative research methods, such as the structural equation model adopted cross-sectional study (27, 71) and the multilevel linear regression adopted in longitudinal study (5). This present cross-sectional study adopted the structural equation model in Amos 21.0 based on maximum likelihood estimates to test if the theoretical pathways (Figure 1) fit the elderly population in Chinese context and explore the pathways between neighborhood greenspaces and older adults' physical and mental health.

To evaluate the reliability of questionnaire data, we conducted reliability analysis on the same type of questionnaire data by using SPSS 21.0. Cronbach's alpha coefficients of social interaction and physical and mental health status as calculated by SPSS were 0.738, 0.912, and 0.939, respectively, indicating that similar questions in the questionnaire had high consistency, good reliability, and substantial research value. In terms of validity, the Kaiser-Meyer-Olkin (KMO) value of the selected data was 0.904 (greater than 0.9) and thus passed the Bartlett sphericity test at the 99.9% confidence level, suggesting that the selected questionnaire data structure had good validity.

## Results

### Descriptions of the Study Population and Greenery Measures

The characteristics of the neighborhoods and study populations are summarized in Table 2 without any missing value. Almost half of the respondents were male (43.1%), and 78.1% were young seniors (60–74 years old). About one third (31.9%) of the respondents with the monthly income below 2,100 yuan (according to the minimum wage standard in Guangzhou) belong to low-income group; 77.2% were married, and 69.0% had consistent registered residence status (hukou) with their living address, which means they are local residents.

**Table 2:** Summary statistics for all studied variables.

**Variables**	**Proportion/Mean (Standard Deviation)**
***Population characteristics (total population=972)***	
**Gender (%)**	
Male	43.1%
Female	56.9%
**Age**	
60–74 years old	78.1%
=75 years old and above	28.2%
**Estimate monthly income (%)**	
0–2,100 yuan (low income)	31.9%
2 = 2,100 yuan and above (median or high income)	68.1%
**Marital status (%)**	
Married	77.2%
Single, divorced or widowed	22.8%
**Registered residence status (HUKOU)**	
Local registered resident	69.0%
Nonlocal registered resident	31.0%
***Predictors***	
X1 Neighborhood streetscape greenery median (q25-q75)	0.174 (0.110–0.378)
X2 Neighborhood (NDVI) median (q25-q75)	0.134 (0.108–0.190)
***Mediators***	
Y1 physical activity(hour)	1.544 (1.056)
Social cohesion	3.806 (0.790)
Y2 I think that I know many people in the community (1–5)	3.708 (0.978)
Y3 I am willing to communicate with community members (1–5)	3.903 (0.777)
Y4 neighborhood PM2.5 median (q25-q75)	61.690 (47.023 – 75.760)
***Outcome***	
Physical health (1–5)	3.421 (0.855)
Y5 I seem to get sick easier than others (1–5)	3.468 (1.072)
Y6 I have poor health condition (1–5)	3.484 (1.051)
Y7 Feel hard to do heavy exercise activities (such as running, playing, lifting weights, etc.) (1–5)	2.842 (1.221)
Y8 Feel hard to do moderate exercise activities (such as lifting tables, cleaning rooms, doing gymnastics, etc.) (1–5)	3.365 (1.175)
Y9 Feel hard to climb the stairs (1–5)	3.170 (1.228)
Y10 Feel hard to bend and kneel (1–5)	3.295 (1.207)
Y11 Feel hard to walk for about 20 minutes (1–5)	3.670 (1.096)
Y12 Feel hard to bathing and dressing yourself (1–5)	3.997 (0.925)
Y13 Has your body been in pain (such as headache, chest tightness, nausea, etc.) in the past four weeks? (1–5)	3.371 (1.236)
Y14 Has the physical pain affected your work and housework in the past for weeks?(1–5)	3.549 (1.180)
Mental Health (1–5)	3.950 (0.754)
Y15 I feel I am in good mental health status (1–5)	3.985 (0.814)
Y16 I feel calm (1–5)	4.042 (0.803)
Y17 I feel good and happy (1–5)	4.021 (0.875)
Y18 I can concentrate on the things that I am doing (1–5)	3.984 (0.853)
Y19 I don't feel stressed (1–5)	3.880 (1.040)
Y20 I am not nervous (1–5)	3.951 (0.932)
Y21 I don't feel downcast and nothing can cheer me up (1–5)	3.939 (0.922)
Y22 I feel energetic (1–5)	3.797 (0.933)

**Figure 3:**
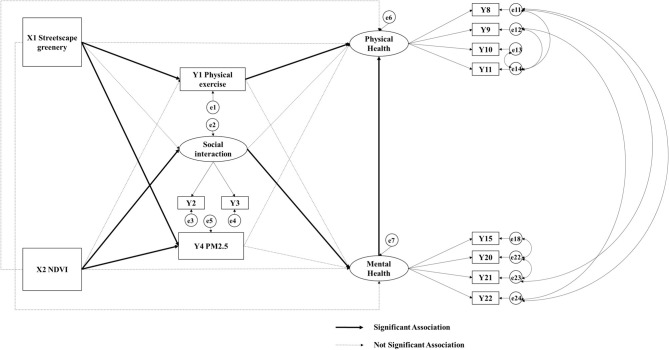
Modified structural equation model and results.

The median scores for neighborhood streetscape greenery and NDVI were 0.174 and 0.134, respectively. No statistical correlation was observed between these variables (*r* = 0.035, *p* = 0.4314), which justifies using them as two separate observable indicators in the structural equation model (Figure 3). The standard deviation (SD) and 25–75 quantile represent variation and the dispersion degree of the data. In terms of mediators, the average time spent on physical exercise of all respondents are about one and half hours, with a standard deviation of 1.056 h, indicating a relatively large variance. The average social interaction score and the median neighborhood PM_2.5_ concentration were 3.806 (SD = 0.790) and 61.690 μg/m^3^ respectively, which is higher than WHO air quality guideline for PM_2.5_ 24-h concentrations (25 μg/m^3^) (74). With regard to health outcomes, the average scores of physical and mental health were 3.421 (SD: 0.855) and 3.950 (SD: 0.754), respectively, indicating relatively good overall health status among the respondents.

### Model Modifications and Fit

The RMSEA (Root Mean Square Error of Approximation) value of the initial theoretical structural equation model was exceptionally high. On the basis of the revised index MI and *t* values suggested by the Amos software, modifications of the model were made separately and once a time. The model was analyzed to determine whether the corrections were reasonable by comparing the model fitness index and the Chi-square value before and after the corrections and by ensuring that the model had practical theoretical importance. The modifications involved increasing notable impact paths, such as the impact path between mental and physical health, and deleting observation variables and their paths that do not make a meaningful contribution and remained the loads of the observed variable loading factors of mental and physical health were higher than 0.71. After modifications, the SEM showed a sufficiently good fit to the data: GFI (Goodness-of-fit index) = 0.978 (>0.9) and RMSEA = 0.039 (<0.05). The results and modification are shown in Table 3 and Figure 3.

**Table 3:** Standardized estimates and the significance level of modified structural equation model.

**Pathways**	**Standardized estimates**	***P-*value**
**Mediating pathway via physical exercise**		
Streetscape greenery → Physical exercise	0.18	^***^
NDVI → Physical exercise	−0.006	0.054
Physical exercise → Physical health	0.15	^***^
Physical exercise → Mental health	0.03	0.390
**Mediating pathway via social interaction**		
NDVI → Social interaction	0.08	0.033^*^
Streetscape greenery → Social interaction	0.04	0.33
Social interaction → Mental health	0.17	^***^
Social interaction → Physical health	0.00	0.960
**Mediating pathway via PM 2.5**		
Streetscape greenery → PM 2.5	−0.39	^***^
NDVI → PM 2.5	−0.40	^***^
PM 2.5 → Physical health	0.03	0.853
PM 2.5 → Mental health	0.01	0.831
**Direct association**		
Streetscape greenery → Physical health	0.00	0.895
Streetscape greenery → Mental health	0.03	0.438
NDVI → Physical health	−0.06	0.053
NDVI → Mental health	−0.04	0.334
**New pathway: association between physical health and mental health**		
Physical health → Mental health	0.46	^***^

* *Means significant at 95% confidence interval*.

*** *Means significant at 99.9% confidence interval*.

### Model Results

In terms of direct relationships, neighborhood NDVI was not statistically significantly associated with older adults' neither the physical nor mental health at the 95% confidence level. With regard to mediating associations, neighborhood streetscape greenery was positively related to older adults' average time spent on physical activity but negatively related to neighborhood PM_2.5_ concentrations at the 99.9% confidence interval. Neighborhood NDVI was positively related to older adults' social interaction at the 95% confidence interval but negatively related to neighborhood PM_2.5_ concentrations at the 99.9% confidence interval. Older adults' physical activity and level of social interaction were positively associated with their physical and mental health, respectively, at the 99.9% confidence level. Moreover, older adults' mental health was positively related to their physical health at the 99.9% confidence level.

The significant positive association pathways which are consistent with hypothesis includes “streetscape greenery—physical exercise—physical health,” “neighborhood NDVI—social interaction—mental health,” and the positive association between mental and physical health is newly found. The next step is to analyze the difference of these association pathways among elderly individuals with various socio-demographic characteristics.

### Multigroup Analysis

**Figure 4:**
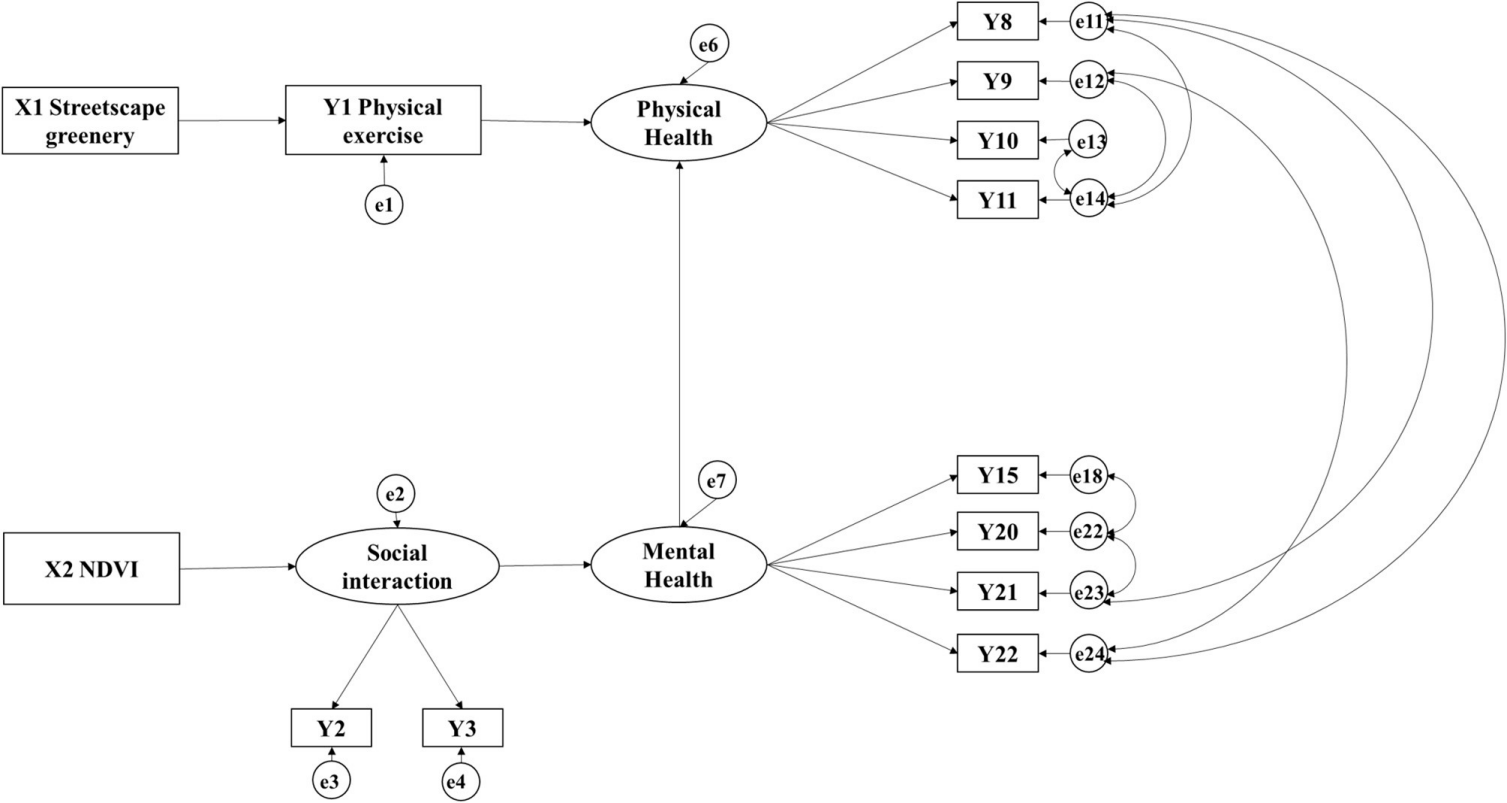
Structural equation model used for multigroup analysis.

On the basis of the SEM developed above, which is applicable to the entire older adults' group, the less significant (*p* > 0.05) pathways were deleted with only meaningful pathways remained (Figure 4). Control variables of different incomes, gender, marital status and registered residence status (hukou) were grouped as the same criteria (Table 4) to perform multigroup SEM analysis (Multi-Group Analysis) and further explore differences in the pathways between two corresponding groups.

**Table 4:** Standardized estimates and the significance level of unconstrained model of multigroup analyses.

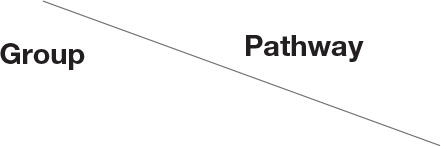		**Streetscape greenery→ Physical exercise**	**Physical exercise→ Physical health**	**NDVI→ Social interaction**	**Social interaction→ Mental health**	**Mental health→ Physical health**
Estimate Monthly income	0-2100yuan	0.16^**^	0.17^**^	0.16^***^	**0.01**	0.38^***^
	2100 and above (medium and high income)	0.20^***^	0.14^***^	**0.04**	0.17^**^	0.47^***^
Gender	Male	0.19^***^	**0.08**	0.24^***^	**0.11**	0.50^***^
	Female	0.20^***^	0.20^***^	**−0.08**	0.21^**^	0.43^***^
Marital status	Married	0.16^***^	0.14^***^	0.13^**^	0.12^*^	0.49^***^
	Single, divorced or widowed	0.29^***^	0.19^**^	**-0.06**	**0.19**	0.35^***^
Registered residence status (Hukou)	Local registered resident:	0.12^**^	0.16^***^	0.04	0.18^***^	0.44^***^
	Non-local registered resident	0.23^***^	0.14^**^	0.07	**0.1**	0.50^***^

* *Means significant at 95% confidence interval*.

** *Means significant at 99% confidence interval*.

*** *Means significant at 99.9% confidence interval*.

*Bold means the corresponding pathways are considered significantly different*.

Four models, namely, unconstrained model, measurement weights restricted model, structural weights restricted model, and measurement residuals restricted model, were calculated and fitted well (GFI > 0.9, RMESA < 0.05). A significant difference (*p* < 0.05) in Chi-square value between the unrestricted and measurement residuals restricted models denotes differences between two corresponding groups. The Chi-square value of the unrestricted and restricted models significantly increased (*p* < 0.05) regarding income, gender, marital status, and registered residence status (hukou), indicating that these variables had a significant regulating effect.

In comparing the pathways between two corresponding groups of significant difference, critical ratios for differences between parameters are used for comparison when both paths are significant. When the critical ratio for difference between parameters was <1.96 (at the 95% confidence interval and higher), the two corresponding pathways were considered equal and vice versa. If one pathway is significant (*p* < 0.05) while the corresponding one is not (*p* ≥ 0.05), these pathways are considered different. If two corresponding pathways are considered different in either way mentioned above, they are marked in bold in Table 4. In terms of income, the results showed that the level of social interactions of individuals belonging to low-income groups was more strongly associated with neighborhood NDVI, but it had no association with their mental health status. In terms of gender, the physical and mental health of female older adults were more significantly related to average time spent on physical exercise and level of social interaction than that of male elderly. With regard to marital status, the level of social interaction of married older adults showed a significant relationship with neighborhood greenery (at the 99% confidence interval) and significant linkage to mental health (at the 95% confidence interval). By contrast, the social interaction level in unmarried older adults' group showed neither significant association with neighborhood greenery indicators nor exhibited significant association with their mental health. With regard to registered residence status (hukou), the level of social interaction of local groups showed no significant relationship with neighborhood greenery metrics but exhibited significant association with their mental health. Among non-local groups, the level of social interaction was neither significantly positively linked to neighborhood streetscape greenery nor to their mental health.

## Discussion

Consistent with previous studies, the present study confirmed that the mediating pathways where neighborhood greenspaces have a positive relationship physical and mental health via physical exercise and social interactions, respectively (55, 60, 75). By adopting the research methods including establishing a theoretical SEM on the basis of the results of previous studies and modifying it accordingly to achieve good model fit. We used the modified model to explore and analyze the internal logical relationships and pathways between greenspaces and the physical and mental health of the older adults in 20 residential neighborhoods in China. We conducted a multigroup analysis to explore whether and how the relationships between neighborhood greenspaces and the well-being of the older adults was different among five control groups. The results of the present study extend the knowledge on this topic in the following aspects. First, this study was the first to systematically investigate the pathways that link neighborhood greenspaces and the physical and mental health of the older adults in a densely populated Chinese city. Second, this study investigated differences in pathways among various control groups of older adults. Third, the study made a methodological contribution by adopting both bird's-eye view NDVI and human-scale streetscape greenery to measure neighborhood greenness.

### The Association Between Neighborhood Greenspace and Older Adults' Well-Being

Neighborhood greenspaces are positively related to older adults' physical health via physical activity. Existing research generally agrees that neighborhood greenspaces encourage residents to engage in physical activities (such as walk, run, bike, and other sports) and provide more opportunities for people to exercise, thereby increasing the average time they spend on physical activities (60). Numerous studies conducted in developed countries support the positive association between greenspaces and physical activity among adults (76–78) and children (75). For example, a study in the UK suggested the urban greenspaces are valuable resources to encourage physical activity among children (75). A study in Europe found that large expanses of greenery in residential environments promote more physical activities among adults (77). The present study expands these conclusions to the order residents possibly because they no longer work and spend more time in their neighborhoods. Hence, the association between neighborhood greenspaces with their level of physical activity is more prominent. Older adults who are willing to do physical exercises often participate in morning exercises, group dancing, and other sports in open neighborhood greenspaces, but those who do not participate in such activities still walk around the neighborhood. Trees beside neighborhood lanes provide shade and make their walk enjoyable, thereby promoting physical exercise. Some suburban seniors still work as farmers, which is also a form of physical exercise. Previous studies also suggested that high levels of physical exercise are associated with good physical and mental health (79, 80). The present study proved this theory in terms of physical health: the longer the older adults exercise frequently has better physical health than those who are mostly sedentary. Studies have shown that physical activities among older adults can preserve muscle mass and reduce age-related decrease in metabolic rate (81), which can potentially result in reducing morbidity and mortality and postponing disability (82). However, the present study found no statistically significant relationships between the elderly's physical activity and their mental health. A possible explanation is that different intensities of physical activity may have a different relationship with older adults' mental health, and intense activity may lead to mood variation and mental deterioration which are more related to the construct of depression (83). However, this study did not consider of the intensity of activity.

Neighborhood greenspaces are positively associated with older adults' mental health via social interactions. Multiple empirical studies in both developed and developing countries have demonstrated that neighborhood greenspaces, as a meeting place for social interactions, have a positive relation to social cohesion (58, 59). Some of these studies were conducted in the Netherlands that covered all age groups above 12 years old (57) and aging populations aged 60 years and older (59). A similar study was conducted in Australia that focused on age groups between 20 and 65 years old (84). Another study was performed in China that concentrated on adults (21). Other works also found a positive relationship between social cohesion and mental health (85) and physical health (62). The present study reaffirmed this pathway to mental health in the context of the older adults in China. In Guangzhou City, most seniors walk together to chat or drink tea almost every day, and the street greenery makes their walks more comfortable, especially during the summer. They meet with friends to acquaint each other in greenspaces where the conditions are cooler. These are places where they play cards and chess. Old tall trees serve as their shade from the sun. The old adults from suburban areas flock in greenspaces to share their experiences at work. Social interactions can positively affect the older adults' perception of their aging status and their own sense of value in their neighborhoods, thereby promoting their mental health (86). Moreover, social interactions can alleviate the older adults' emotional problems through continuous and meaningful interactions with social members. These interactions increase their feelings of positive emotions and thus positively associates with their mental health (87). However, the present study did not find significant linkages between social contacts and physical health among older adults. A possible explanation behind this result is that the definition of social contacts in this study was slightly different from the concept of social cohesion in terms of strength of social ties (88). In addition, social interaction may have detrimental effects, since more interaction may result in more confliction than the counterparts (89).

The hypothesis that neighborhood greenspaces are positively associated with the older adults' physical and mental health by reducing air pollution was not supported because the results showed that neighborhood greenspaces were negatively related to neighborhood PM_2.5_ concentrations, but this parameter exhibited no significant linkage to older adults' physical and mental health. This result contradicted that of several theoretical (10, 90) and empirical studies (71, 91). Especially for older adults, existing studies suggest that PM2.5 concentration is significantly related to their respiratory system (92) and elderly is a susceptible population to PM_2.5_ associated diseases (93–95) and PM_2.5_ related depressive and anxiety symptoms (96). A possible reason for this contradiction is that the association between health and air pollution could only be demonstrated after a relatively long-term exposure (97–99), and certain diseases, including cardiovascular disease, respiratory disease such as asthma, and cancer (92, 100, 101), which takes a while to manifest, while this study is cross-sectional. Moreover, both the mental and physical health status acquired in this study was self-rated. Another possible explanation is that PM_2.5_ concentrations are varying over time. The data obtained in this study only reflected the status of air quality during measurement; thus, the short period of air quality measurement led to bias in data collection.

The present study did not observe strong and significant direct association between neighborhood greenspaces and older adults' well-being. This result contradicted that of previous studies (21). According to the literature, neighborhood greenery is positively related to an individual's physical (102, 103) and mental health (21). A possible reason behind this inconsistency is that the association between short-term effects of exposure to greenspaces and long-term physical and mental health of older adults is not significant. Most existing studies focused on the association between greenspaces and a specific health aspect instead of overall health status. For example, a study that examined short-term changes in vascular risk factors found a positive relationship between urban green environments and health (102). This cross-sectional study had a smaller scope and encompassed a shorter time period. Another possible explanation is that not all plants can have beneficial effects on residents' health, and some plants that are toxic, attract insects, or easily become allergens actually have negative effects on their residents (104).

An unexpected result was obtained by the present study: the mental health of older adults was positively associated with their physical health. An empirical study in Australia showed that positive attitudes to aging are associated with positive self-reported physical health status (105). Older adults with strong mental health status and positive self-recognition are more likely to have excellent physical health.

### The Association Pathway Among Older Adults With Different Sociodemographic Characteristics

We performed a multigroup analysis to explore the differences among older adults with different sociodemographic characteristics. Overall, the “neighborhood greenspace—physical exercise—physical health” pathway is significant in all older adults' group except male group. While the “neighborhood greenspace—social interaction—mental health” pathway is dramatically different among the corresponding groups. The level of social interaction of individuals belonging to low-income group was more significantly associated with neighborhood greenspaces probably because of limited expendable funds they have to pay for social activities outside their neighborhoods and unfamiliarity to their external environment, indicating that they are more dependent on freely accessible neighborhood greenspaces. However, the social interaction parameter was not significantly associated with their mental health status probably results from social exclusion due to their disadvantaged social status. As for female older adults, their mental and physical health are more significantly associated with social interaction physical exercise, respectively, than those of male elderly. While the social interaction level of the male group is more significantly linked to the neighborhood NDVI than the female group, probably because most male older adults communicate with friends within the neighborhood greenspace than female. With regard to unmarried elderly, the association of neighborhood green spaces on their level of social interaction was not significant because their interactions with close friends is not necessarily limited to neighborhood greenspaces and they tend to have more freedom to go outside the neighborhood. In terms of registered residency status, the level of social interaction of the non-local groups was neither significantly related to neighborhood greenery nor to their mental health. A possible explanation is that the social network of these non-local older adults remains in their former residence, and the neighborhood network here seems foreign to them. In general, older adults, especially those who belong to low-income group, have limited spaces for activities and socializing that is why they are more dependent on neighborhood greenspaces, a supposition consistent with that of previous studies (106, 107).

### Strengths and Limitations

The present study was one of the first to examine the association pathways between neighborhood greenspaces and older adults' physical and mental health in a Chinese city. This study has two main strengths. First, we adopted two metrics of neighborhood greenery, namely, bird's-eye view and human view, to minimize statistical bias. Second, we concentrated on older adults with different sociodemographic characteristics to identify differences among groups and focus on vulnerable groups.

This study has several limitations that must be addressed in a future work. First, it is a study based on cross-sectional data and research design, which may overestimate the association make it difficult to infer causation between neighborhood greenspace and older adults' well-being. Second, streetscape and PM_2.5_ data were collected via field surveys and with relatively limited sample sizes. Hence, the data may lack accuracy. Other data acquisition methods, such as calculating the average annual air pollution concentration from secondary data sources (71), extracting street view images from maps (27, 70) and focusing on the respiratory morbidity of older adults, may improve the accuracy and find meaningful result. Third, the health outcomes were determined from subjective questionnaire surveys. Objective health outcomes, such as BMI and morbidity, may be more reliable. Moreover, the greenspace accessibility analysis as well as the association pathways analysis among communities with different housing nature could be also taken into consideration in the future research with larger sample size.

## Conclusions and Recommendations

On the basis of review of literature on greenspaces and residents' well-being, we constructed an SEM linking neighborhood greenspaces and older adults' physical and mental health status. We obtained primary data from 972 urban and rural elderly populations and streetscape greenery and PM_2.5_ concentration data from field surveys of 20 residential neighborhoods in Guangzhou City from 2018 to 2019. We also gathered secondary data from neighborhood committee level NDVI data from 2017 Landsat image. We found that neighborhood greenspaces have a positive relationship with older adults' physical exercise, thereby positively associates with their physical health. Moreover, greenspaces have a positive linkage with older adults' social interaction. Thus, greenspaces are positively associated with their mental health. These findings are consistent with those of previous studies. However, we found that neighborhood greenspaces have no significant direct association with older adults' physical and mental health. Furthermore, we found that the level of social interaction is more significantly related to neighborhood greenspaces among low-income groups, and mental health is more significantly linked to the level of social interaction among local and unmarried groups. Based on the findings, we suggest that urban planners should design neighborhood greenspaces where the older adults can exercise and communicate with each other. They can also plant trees along sidewalks to provide desirable walking environment for seniors, and recreational infrastructures under shaded trees to increase community interactions among the older adults. Finally, they can develop greenspaces in communities with a high proportion of vulnerable elderly, especially for those that belong to low-income groups.

## Data Availability Statement

The datasets presented in this article are not readily available because of institutional copyright issues. Requests to access the datasets should be directed to Yuan Yuan, yuanyuan@mail.sysu.edu.cn.

## Ethics Statement

The studies involving human participants were reviewed and approved by School of Geography and Planning, Sun Yat-sen University. The patients/participants provided their written informed consent to participate in this study.

## Author Contributions

YY contributed to the conception and design of the study and is in charge of the project. YZ, YC, and SL contributed to data preparation, collection, and organization. YZ performed the statistical analysis, structured, and wrote the first draft of the manuscript. YY, YZ, and YC contributed to manuscript revision. All authors read and approved the submitted version.

## Conflict of Interest

The authors declare that the research was conducted in the absence of any commercial or financial relationships that could be construed as a potential conflict of interest.
